# (20*S*)-22-Iodo­methyl-6β-meth­oxy-3α,5-dihydro-3′*H*-cyclo­propa[3α,5]-5α-pregnane

**DOI:** 10.1107/S1600536809014597

**Published:** 2009-04-25

**Authors:** Kamal Aziz Ketuly, A. Hamid A. Hadi, Seik Weng Ng

**Affiliations:** a Department of Chemistry, University of Malaya, 50603 Kuala Lumpur, Malaysia

## Abstract

In the title steroid derivative, C_23_H_37_IO, the fused cyclo­propane unit that comprises part of the *A* ring has a β-configuration, and the associated cyclo­pentane ring has an envelope conformation.

## Related literature

This iodo-substituted steroid was synthesized from 22-(*p*-toluene­sulfonyl­oxymeth­yl)-6β-meth­oxy-3α,5-cyclo-5α-pregnane; for its crystal structure, see: Ketuly *et al.* (1997 ▶).
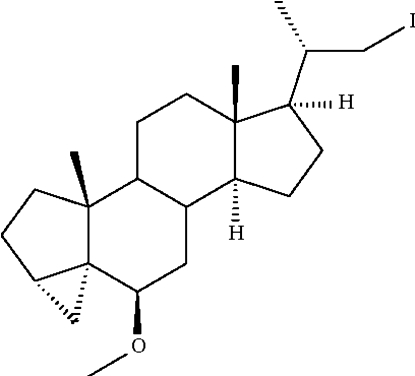


## Experimental

### 

#### Crystal data

C_23_H_37_IO
*M* *_r_* = 456.43
Monoclinic, 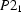

*a* = 7.4001 (1) Å
*b* = 9.8862 (1) Å
*c* = 14.8768 (2) Å
β = 100.593 (1)°
*V* = 1069.82 (2) Å^3^
*Z* = 2
Mo *K*α radiation
μ = 1.51 mm^−1^
*T* = 100 K
0.35 × 0.20 × 0.05 mm

#### Data collection

Bruker SMART APEX diffractometer
Absorption correction: multi-scan (*SADABS*; Sheldrick, 1996 ▶) *T*_min_ = 0.605, *T*_max_ = 0.746 (expected range = 0.752–0.928)
10136 measured reflections
4895 independent reflections
4652 reflections with *I* > 2σ(*I*)
*R*_int_ = 0.021

#### Refinement

*R*[*F* ^2^ > 2σ(*F* ^2^)] = 0.023
*wR*(*F* ^2^) = 0.057
*S* = 0.91
4895 reflections
226 parameters
1 restraint
H-atom parameters constrained
Δρ_max_ = 1.08 e Å^−3^
Δρ_min_ = −0.40 e Å^−3^
Absolute structure: Flack (1983 ▶), 2124 Friedel pairs
Flack parameter: 0.00 (1)

### 

Data collection: *APEX2* (Bruker, 2008 ▶); cell refinement: *SAINT* (Bruker, 2008 ▶); data reduction: *SAINT*; program(s) used to solve structure: *SHELXS97* (Sheldrick, 2008 ▶); program(s) used to refine structure: *SHELXL97* (Sheldrick, 2008 ▶); molecular graphics: *X-SEED* (Barbour, 2001 ▶); software used to prepare material for publication: *publCIF* (Westrip, 2009 ▶).

## Supplementary Material

Crystal structure: contains datablocks global, I. DOI: 10.1107/S1600536809014597/tk2434sup1.cif

Structure factors: contains datablocks I. DOI: 10.1107/S1600536809014597/tk2434Isup2.hkl

Additional supplementary materials:  crystallographic information; 3D view; checkCIF report

## Figures and Tables

**Fig. 1.**
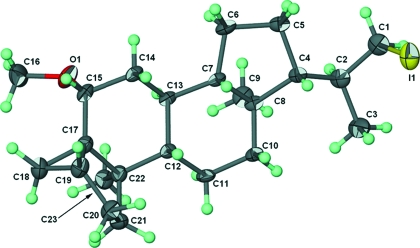
Displacement ellipsoid plot (Barbour, 2001) of C23H37IO at the 70% probability level. H atoms are drawn as spheres of arbitrary radius.

## supplementary crystallographic information

### Experimental

The reactant, 22-(*p*-toluenesulfonyloxymethyl)-6β-methoxy-3α,5-cyclo-5α-pregnane (250 mg, 0.5 mmol) and sodium iodide (756 mg, 5.0 mmol) were dissolved in acetone and the solution heated for 12 h. The solvent was removed and water added. The organic compound was extracted with ethyl acetate. The ethyl acetate was removed to furnish 228 mg of crude product. This was chromatographed through silica gel and eluted with hexane–ethyl acetate (8:1 v/v). The second fraction was a viscous oil (223 mg); this slowly solidified. Single crystals were obtained by vacuum sublimation, m.p. 375–376 K.

### Refinement

Hydrogen atoms were placed at calculated positions (C–H 0.98–1.00 Å) and were treated as riding on their parent carbon atoms, with *U*(H) set to 1.2–1.5 times *U*_eq_(*C*). The final difference Fourier map had a peak/hole in the vicinity of the iodide atom.

### Crystal data

**Table:**

C_23_H_37_IO	*F*(000) = 472
*M**_r_* = 456.43	*D*_x_ = 1.417 Mg m^−^^3^
Monoclinic, *P*2_1_	Mo *K*α radiation, λ = 0.71073 Å
Hall symbol: P 2yb	Cell parameters from 7037 reflections
*a* = 7.4001 (1) Å	θ = 2.5–28.3°
*b* = 9.8862 (1) Å	µ = 1.51 mm^−^^1^
*c* = 14.8768 (2) Å	*T* = 100 K
β = 100.593 (1)°	Wedge, colorless
*V* = 1069.82 (2) Å^3^	0.35 × 0.20 × 0.05 mm
*Z* = 2	

### Data collection

**Table:**

Bruker SMART APEX diffractometer	4895 independent reflections
Radiation source: fine-focus sealed tube	4652 reflections with *I* > 2σ(*I*)
graphite	*R*_int_ = 0.021
ω scans	θ_max_ = 27.5°, θ_min_ = 1.4°
Absorption correction: multi-scan (*SADABS*; Sheldrick, 1996)	*h* = −9→9
*T*_min_ = 0.605, *T*_max_ = 0.746	*k* = −12→12
10136 measured reflections	*l* = −18→19

### Refinement

**Table:**

Refinement on *F*^2^	Secondary atom site location: difference Fourier map
Least-squares matrix: full	Hydrogen site location: inferred from neighbouring sites
*R*[*F*^2^ > 2σ(*F*^2^)] = 0.023	H-atom parameters constrained
*wR*(*F*^2^) = 0.057	*w* = 1/[σ^2^(*F*_o_^2^) + (0.0354*P*)^2^ + 0.1213*P*] where *P* = (*F*_o_^2^ + 2*F*_c_^2^)/3
*S* = 0.91	(Δ/σ)_max_ = 0.001
4895 reflections	Δρ_max_ = 1.08 e Å^−^^3^
226 parameters	Δρ_min_ = −0.40 e Å^−^^3^
1 restraint	Absolute structure: Flack (1983), 2124 Friedel pairs
Primary atom site location: structure-invariant direct methods	Flack parameter: 0.00 (1)

### Fractional atomic coordinates and isotropic or equivalent isotropic displacement parameters (Å^2^)

**Table:**

	*x*	*y*	*z*	*U*_iso_*/*U*_eq_	
I1	0.49950 (2)	0.50001 (3)	0.779194 (9)	0.03191 (5)	
O1	0.4077 (3)	0.27281 (19)	0.11997 (13)	0.0285 (4)	
C1	0.4448 (4)	0.2961 (3)	0.72899 (18)	0.0265 (5)	
H1A	0.5622	0.2548	0.7202	0.032*	
H1B	0.3986	0.2429	0.7764	0.032*	
C2	0.3067 (3)	0.2842 (2)	0.63954 (17)	0.0233 (5)	
H2	0.2940	0.1854	0.6254	0.028*	
C3	0.1162 (4)	0.3333 (3)	0.6515 (2)	0.0348 (6)	
H3A	0.0803	0.2868	0.7037	0.052*	
H3B	0.0265	0.3133	0.5960	0.052*	
H3C	0.1199	0.4310	0.6625	0.052*	
C4	0.3755 (3)	0.3500 (2)	0.55863 (16)	0.0178 (4)	
H4	0.3838	0.4495	0.5706	0.021*	
C5	0.5707 (3)	0.3009 (2)	0.54875 (17)	0.0203 (5)	
H5A	0.6658	0.3619	0.5824	0.024*	
H5B	0.5932	0.2083	0.5736	0.024*	
C6	0.5768 (3)	0.3026 (3)	0.44530 (17)	0.0221 (5)	
H6A	0.6860	0.3525	0.4333	0.027*	
H6B	0.5794	0.2096	0.4210	0.027*	
C7	0.3996 (3)	0.3752 (2)	0.40278 (15)	0.0155 (4)	
H7	0.4223	0.4735	0.4163	0.019*	
C8	0.2589 (3)	0.3300 (2)	0.46145 (16)	0.0168 (4)	
C9	0.2055 (3)	0.1797 (2)	0.44589 (18)	0.0241 (5)	
H9A	0.1182	0.1546	0.4852	0.036*	
H9B	0.3159	0.1234	0.4609	0.036*	
H9C	0.1485	0.1658	0.3817	0.036*	
C10	0.0911 (3)	0.4223 (2)	0.43631 (16)	0.0188 (5)	
H10A	−0.0048	0.3943	0.4710	0.023*	
H10B	0.1267	0.5166	0.4536	0.023*	
C11	0.0133 (3)	0.4157 (2)	0.33377 (16)	0.0198 (5)	
H11A	−0.0922	0.4785	0.3196	0.024*	
H11B	−0.0330	0.3231	0.3182	0.024*	
C12	0.1541 (3)	0.4519 (2)	0.27403 (15)	0.0162 (4)	
H12	0.1914	0.5476	0.2893	0.019*	
C13	0.3311 (3)	0.3658 (2)	0.29976 (15)	0.0163 (4)	
H13	0.3017	0.2693	0.2829	0.020*	
C14	0.4773 (3)	0.4161 (2)	0.24774 (17)	0.0191 (4)	
H14A	0.5117	0.5099	0.2671	0.023*	
H14B	0.5885	0.3591	0.2638	0.023*	
C15	0.4129 (3)	0.4134 (2)	0.14454 (16)	0.0203 (5)	
H15	0.5056	0.4607	0.1146	0.024*	
C16	0.4180 (4)	0.2496 (3)	0.02721 (19)	0.0359 (7)	
H16A	0.4122	0.1522	0.0149	0.054*	
H16B	0.5341	0.2859	0.0146	0.054*	
H16C	0.3148	0.2948	−0.0121	0.054*	
C17	0.2296 (3)	0.4825 (3)	0.11759 (14)	0.0199 (5)	
C18	0.1735 (4)	0.5323 (2)	0.01961 (17)	0.0261 (6)	
H18A	0.2623	0.5201	−0.0221	0.031*	
H18B	0.0427	0.5230	−0.0098	0.031*	
C19	0.2204 (4)	0.6337 (3)	0.09593 (17)	0.0240 (5)	
H19	0.3374	0.6863	0.1017	0.029*	
C20	0.0609 (4)	0.6938 (3)	0.1333 (2)	0.0260 (6)	
H20A	−0.0077	0.7597	0.0898	0.031*	
H20B	0.1044	0.7396	0.1925	0.031*	
C21	−0.0598 (3)	0.5708 (3)	0.14540 (17)	0.0229 (5)	
H21A	−0.1483	0.5526	0.0882	0.027*	
H21B	−0.1295	0.5874	0.1952	0.027*	
C22	0.0730 (3)	0.4498 (2)	0.16951 (16)	0.0189 (4)	
C23	−0.0222 (4)	0.3158 (3)	0.13919 (19)	0.0249 (5)	
H23A	0.0645	0.2411	0.1560	0.037*	
H23B	−0.0640	0.3166	0.0727	0.037*	
H23C	−0.1282	0.3039	0.1694	0.037*	

### Atomic displacement parameters (Å^2^)

**Table:**

	*U*^11^	*U*^22^	*U*^33^	*U*^12^	*U*^13^	*U*^23^
I1	0.04946 (10)	0.02171 (7)	0.02581 (8)	−0.00363 (10)	0.01026 (6)	0.00349 (9)
O1	0.0350 (10)	0.0228 (9)	0.0314 (10)	0.0019 (7)	0.0159 (8)	−0.0082 (8)
C1	0.0330 (13)	0.0179 (12)	0.0293 (13)	0.0001 (10)	0.0075 (11)	0.0068 (10)
C2	0.0240 (12)	0.0204 (12)	0.0264 (12)	−0.0007 (9)	0.0070 (10)	0.0073 (10)
C3	0.0265 (13)	0.0503 (18)	0.0310 (14)	0.0035 (12)	0.0141 (11)	0.0173 (13)
C4	0.0161 (10)	0.0155 (10)	0.0222 (11)	0.0004 (8)	0.0047 (8)	0.0032 (8)
C5	0.0161 (10)	0.0167 (11)	0.0281 (12)	0.0010 (8)	0.0040 (9)	0.0031 (9)
C6	0.0156 (10)	0.0223 (12)	0.0301 (13)	0.0035 (9)	0.0085 (9)	0.0004 (10)
C7	0.0121 (9)	0.0142 (10)	0.0215 (11)	−0.0001 (8)	0.0062 (8)	−0.0012 (8)
C8	0.0137 (9)	0.0134 (10)	0.0241 (11)	−0.0021 (8)	0.0057 (8)	0.0006 (8)
C9	0.0254 (12)	0.0174 (11)	0.0298 (13)	−0.0069 (9)	0.0063 (10)	−0.0003 (10)
C10	0.0128 (10)	0.0220 (12)	0.0227 (12)	0.0015 (8)	0.0059 (8)	0.0000 (9)
C11	0.0107 (10)	0.0243 (12)	0.0247 (12)	0.0024 (8)	0.0042 (9)	0.0018 (9)
C12	0.0138 (9)	0.0169 (9)	0.0185 (11)	−0.0002 (7)	0.0045 (8)	−0.0011 (8)
C13	0.0128 (9)	0.0148 (10)	0.0221 (11)	−0.0003 (8)	0.0052 (8)	−0.0041 (8)
C14	0.0144 (10)	0.0213 (12)	0.0232 (11)	−0.0006 (8)	0.0075 (8)	−0.0032 (9)
C15	0.0196 (11)	0.0209 (11)	0.0226 (12)	−0.0004 (9)	0.0093 (9)	−0.0053 (9)
C16	0.0444 (17)	0.0369 (15)	0.0239 (14)	0.0115 (13)	−0.0005 (12)	−0.0120 (11)
C17	0.0191 (9)	0.0223 (16)	0.0195 (9)	−0.0019 (10)	0.0063 (7)	−0.0023 (10)
C18	0.0302 (12)	0.0283 (17)	0.0206 (11)	−0.0009 (9)	0.0071 (9)	−0.0012 (9)
C19	0.0279 (12)	0.0218 (11)	0.0243 (12)	−0.0006 (9)	0.0099 (10)	−0.0002 (10)
C20	0.0295 (14)	0.0219 (13)	0.0287 (14)	0.0022 (11)	0.0111 (12)	0.0036 (10)
C21	0.0190 (12)	0.0274 (14)	0.0229 (12)	0.0043 (10)	0.0053 (9)	0.0031 (10)
C22	0.0177 (11)	0.0217 (10)	0.0176 (11)	−0.0015 (8)	0.0040 (9)	−0.0014 (8)
C23	0.0217 (12)	0.0286 (14)	0.0239 (13)	−0.0046 (11)	0.0029 (11)	−0.0042 (10)

### Geometric parameters (Å, °)

**Table:**

I1—C1	2.162 (3)	C11—H11A	0.9900
O1—C16	1.415 (3)	C11—H11B	0.9900
O1—C15	1.436 (3)	C12—C13	1.551 (3)
C1—C2	1.526 (4)	C12—C22	1.561 (3)
C1—H1A	0.9900	C12—H12	1.0000
C1—H1B	0.9900	C13—C14	1.525 (3)
C2—C3	1.532 (3)	C13—H13	1.0000
C2—C4	1.535 (3)	C14—C15	1.522 (3)
C2—H2	1.0000	C14—H14A	0.9900
C3—H3A	0.9800	C14—H14B	0.9900
C3—H3B	0.9800	C15—C17	1.506 (3)
C3—H3C	0.9800	C15—H15	1.0000
C4—C5	1.556 (3)	C16—H16A	0.9800
C4—C8	1.554 (3)	C16—H16B	0.9800
C4—H4	1.0000	C16—H16C	0.9800
C5—C6	1.548 (3)	C17—C18	1.522 (3)
C5—H5A	0.9900	C17—C19	1.528 (4)
C5—H5B	0.9900	C17—C22	1.540 (3)
C6—C7	1.527 (3)	C18—C19	1.507 (3)
C6—H6A	0.9900	C18—H18A	0.9900
C6—H6B	0.9900	C18—H18B	0.9900
C7—C13	1.526 (3)	C19—C20	1.516 (3)
C7—C8	1.542 (3)	C19—H19	1.0000
C7—H7	1.0000	C20—C21	1.539 (4)
C8—C10	1.531 (3)	C20—H20A	0.9900
C8—C9	1.544 (3)	C20—H20B	0.9900
C9—H9A	0.9800	C21—C22	1.548 (3)
C9—H9B	0.9800	C21—H21A	0.9900
C9—H9C	0.9800	C21—H21B	0.9900
C10—C11	1.530 (3)	C22—C23	1.529 (4)
C10—H10A	0.9900	C23—H23A	0.9800
C10—H10B	0.9900	C23—H23B	0.9800
C11—C12	1.531 (3)	C23—H23C	0.9800
			
C16—O1—C15	113.7 (2)	C11—C12—H12	106.2
C2—C1—I1	115.24 (16)	C13—C12—H12	106.2
C2—C1—H1A	108.5	C22—C12—H12	106.2
I1—C1—H1A	108.5	C7—C13—C14	110.75 (18)
C2—C1—H1B	108.5	C7—C13—C12	108.88 (17)
I1—C1—H1B	108.5	C14—C13—C12	109.95 (18)
H1A—C1—H1B	107.5	C7—C13—H13	109.1
C1—C2—C3	110.9 (2)	C14—C13—H13	109.1
C1—C2—C4	112.66 (19)	C12—C13—H13	109.1
C3—C2—C4	113.7 (2)	C15—C14—C13	112.72 (18)
C1—C2—H2	106.3	C15—C14—H14A	109.0
C3—C2—H2	106.3	C13—C14—H14A	109.0
C4—C2—H2	106.3	C15—C14—H14B	109.0
C2—C3—H3A	109.5	C13—C14—H14B	109.0
C2—C3—H3B	109.5	H14A—C14—H14B	107.8
H3A—C3—H3B	109.5	O1—C15—C17	113.0 (2)
C2—C3—H3C	109.5	O1—C15—C14	105.24 (19)
H3A—C3—H3C	109.5	C17—C15—C14	111.13 (18)
H3B—C3—H3C	109.5	O1—C15—H15	109.1
C2—C4—C5	112.93 (19)	C17—C15—H15	109.1
C2—C4—C8	117.91 (18)	C14—C15—H15	109.1
C5—C4—C8	103.81 (18)	O1—C16—H16A	109.5
C2—C4—H4	107.2	O1—C16—H16B	109.5
C5—C4—H4	107.2	H16A—C16—H16B	109.5
C8—C4—H4	107.2	O1—C16—H16C	109.5
C6—C5—C4	106.72 (18)	H16A—C16—H16C	109.5
C6—C5—H5A	110.4	H16B—C16—H16C	109.5
C4—C5—H5A	110.4	C15—C17—C18	118.38 (19)
C6—C5—H5B	110.4	C15—C17—C19	120.2 (2)
C4—C5—H5B	110.4	C18—C17—C19	59.22 (17)
H5A—C5—H5B	108.6	C15—C17—C22	119.7 (2)
C7—C6—C5	103.72 (17)	C18—C17—C22	116.76 (18)
C7—C6—H6A	111.0	C19—C17—C22	107.6 (2)
C5—C6—H6A	111.0	C19—C18—C17	60.60 (17)
C7—C6—H6B	111.0	C19—C18—H18A	117.7
C5—C6—H6B	111.0	C17—C18—H18A	117.7
H6A—C6—H6B	109.0	C19—C18—H18B	117.7
C13—C7—C6	119.24 (18)	C17—C18—H18B	117.7
C13—C7—C8	114.63 (18)	H18A—C18—H18B	114.8
C6—C7—C8	104.30 (18)	C18—C19—C20	116.8 (2)
C13—C7—H7	105.9	C18—C19—C17	60.18 (15)
C6—C7—H7	105.9	C20—C19—C17	108.3 (2)
C8—C7—H7	105.9	C18—C19—H19	119.1
C10—C8—C9	111.27 (18)	C20—C19—H19	119.1
C10—C8—C7	106.90 (18)	C17—C19—H19	119.1
C9—C8—C7	112.00 (19)	C19—C20—C21	104.0 (2)
C10—C8—C4	116.00 (18)	C19—C20—H20A	111.0
C9—C8—C4	110.17 (19)	C21—C20—H20A	111.0
C7—C8—C4	99.95 (16)	C19—C20—H20B	111.0
C8—C9—H9A	109.5	C21—C20—H20B	111.0
C8—C9—H9B	109.5	H20A—C20—H20B	109.0
H9A—C9—H9B	109.5	C20—C21—C22	106.28 (19)
C8—C9—H9C	109.5	C20—C21—H21A	110.5
H9A—C9—H9C	109.5	C22—C21—H21A	110.5
H9B—C9—H9C	109.5	C20—C21—H21B	110.5
C11—C10—C8	110.97 (18)	C22—C21—H21B	110.5
C11—C10—H10A	109.4	H21A—C21—H21B	108.7
C8—C10—H10A	109.4	C23—C22—C17	112.5 (2)
C11—C10—H10B	109.4	C23—C22—C21	111.27 (19)
C8—C10—H10B	109.4	C17—C22—C21	103.0 (2)
H10A—C10—H10B	108.0	C23—C22—C12	112.2 (2)
C10—C11—C12	113.42 (18)	C17—C22—C12	108.04 (17)
C10—C11—H11A	108.9	C21—C22—C12	109.40 (18)
C12—C11—H11A	108.9	C22—C23—H23A	109.5
C10—C11—H11B	108.9	C22—C23—H23B	109.5
C12—C11—H11B	108.9	H23A—C23—H23B	109.5
H11A—C11—H11B	107.7	C22—C23—H23C	109.5
C11—C12—C13	111.24 (18)	H23A—C23—H23C	109.5
C11—C12—C22	113.39 (18)	H23B—C23—H23C	109.5
C13—C12—C22	113.05 (18)		
			
I1—C1—C2—C3	−64.1 (2)	C16—O1—C15—C17	80.7 (3)
I1—C1—C2—C4	64.7 (2)	C16—O1—C15—C14	−157.8 (2)
C1—C2—C4—C5	51.6 (3)	C13—C14—C15—O1	−71.9 (2)
C3—C2—C4—C5	178.9 (2)	C13—C14—C15—C17	50.8 (3)
C1—C2—C4—C8	172.75 (19)	O1—C15—C17—C18	−82.4 (3)
C3—C2—C4—C8	−59.9 (3)	C14—C15—C17—C18	159.5 (2)
C2—C4—C5—C6	147.8 (2)	O1—C15—C17—C19	−151.4 (2)
C8—C4—C5—C6	19.0 (2)	C14—C15—C17—C19	90.5 (2)
C4—C5—C6—C7	9.6 (2)	O1—C15—C17—C22	71.3 (3)
C5—C6—C7—C13	−164.53 (19)	C14—C15—C17—C22	−46.7 (3)
C5—C6—C7—C8	−35.1 (2)	C15—C17—C18—C19	−110.1 (3)
C13—C7—C8—C10	−60.0 (2)	C22—C17—C18—C19	95.4 (3)
C6—C7—C8—C10	167.79 (18)	C17—C18—C19—C20	−96.6 (2)
C13—C7—C8—C9	62.1 (2)	C15—C17—C19—C18	107.1 (2)
C6—C7—C8—C9	−70.1 (2)	C22—C17—C19—C18	−111.1 (2)
C13—C7—C8—C4	178.74 (18)	C15—C17—C19—C20	−142.0 (2)
C6—C7—C8—C4	46.6 (2)	C18—C17—C19—C20	110.9 (2)
C2—C4—C8—C10	80.4 (2)	C22—C17—C19—C20	−0.2 (3)
C5—C4—C8—C10	−153.86 (18)	C18—C19—C20—C21	45.7 (3)
C2—C4—C8—C9	−47.1 (3)	C17—C19—C20—C21	−19.4 (3)
C5—C4—C8—C9	78.6 (2)	C19—C20—C21—C22	32.1 (3)
C2—C4—C8—C7	−165.15 (19)	C15—C17—C22—C23	−78.5 (3)
C5—C4—C8—C7	−39.4 (2)	C18—C17—C22—C23	75.7 (3)
C9—C8—C10—C11	−65.6 (2)	C19—C17—C22—C23	139.5 (2)
C7—C8—C10—C11	57.0 (2)	C15—C17—C22—C21	161.6 (2)
C4—C8—C10—C11	167.43 (18)	C18—C17—C22—C21	−44.3 (3)
C8—C10—C11—C12	−56.8 (3)	C19—C17—C22—C21	19.6 (2)
C10—C11—C12—C13	53.2 (3)	C15—C17—C22—C12	45.9 (3)
C10—C11—C12—C22	−178.09 (19)	C18—C17—C22—C12	−160.0 (2)
C6—C7—C13—C14	−57.0 (3)	C19—C17—C22—C12	−96.2 (2)
C8—C7—C13—C14	178.40 (17)	C20—C21—C22—C23	−152.6 (2)
C6—C7—C13—C12	−177.97 (19)	C20—C21—C22—C17	−31.9 (2)
C8—C7—C13—C12	57.4 (2)	C20—C21—C22—C12	82.9 (2)
C11—C12—C13—C7	−51.4 (2)	C11—C12—C22—C23	−53.4 (3)
C22—C12—C13—C7	179.68 (18)	C13—C12—C22—C23	74.4 (2)
C11—C12—C13—C14	−172.89 (19)	C11—C12—C22—C17	−177.9 (2)
C22—C12—C13—C14	58.2 (2)	C13—C12—C22—C17	−50.1 (3)
C7—C13—C14—C15	−177.95 (19)	C11—C12—C22—C21	70.6 (2)
C12—C13—C14—C15	−57.6 (2)	C13—C12—C22—C21	−161.57 (18)
